# Sub-chronic oral toxicity screening of quercetin in mice

**DOI:** 10.1186/s12906-022-03758-z

**Published:** 2022-10-23

**Authors:** Patrice Cunningham, Emma Patton, Brandon N. VanderVeen, Christian Unger, Ahmed Aladhami, Reilly T. Enos, Sarah Madero, Ioulia Chatzistamou, Daping Fan, E. Angela Murphy, Kandy T. Velázquez

**Affiliations:** 1 Department of Pathology, Microbiology, and Immunology, School of Medicine, University of South Carolina, 6439 Garners Ferry Rd., Columbia, SC 29209 USA; 2 AcePre, LLC, Columbia, SC 29209 USA; 3 Department of Cell Biology and Anatomy, School of Medicine, University of South Carolina, Columbia, SC 29209 USA

**Keywords:** Quercetin, Sub-chronic toxicity, Behavior, Metabolism

## Abstract

**Background:**

Quercetin is an organic flavonoid present in several fruits and vegetables. The anti-inflammatory, antiviral, antioxidant, cardio-protective, anti-carcinogenic and neuroprotective properties demonstrated by this dietary supplement endorses it as a possible treatment for inflammatory diseases and cancer. Unfortunately, conflicting research has cast uncertainties on the toxicity of quercetin. The main purpose of this study was to determine if quercetin has any toxic properties in mice at doses that have shown efficacy in pre-clinical studies regarding cancer, cancer therapy, and their off-target effects.

**Methods:**

A sub-chronic toxicity study of quercetin was examined in male and female CD2F1 mice. Three different doses of quercetin (62, 125, and 250 mg/kg of diet) were infused into the AIN-76A purified diet and administered to mice ad libitum for 98 days. Body weight (BW), food consumption, water intake, body composition, blood count, behavior, and metabolic phenotype were assessed at various timepoints during the course of the experiment. Tissue and organs were evaluated for gross pathological changes and plasma was used to measure alkaline phosphatase (AP), aspartate transaminase (AST), and alanine transaminase (ALT).

**Results:**

We found that low (62 mg/kg of diet), medium (125 mg/kg of diet), and high (250 mg/kg of diet) quercetin feeding had no discernible effect on body composition, organ function, behavior or metabolism.

**Conclusions:**

In summary, our study establishes that quercetin is safe for use in both female and male CD2F1 mice when given at ~ 12.5, 25, or 50 mg/kg of BW daily doses for 14 weeks (i.e. 98 days). Further studies will need to be conducted to determine any potential toxicity of quercetin following chronic ingestion.

## Background

Quercetin (3,4,5,7-pentahydroxylflavone) is an organic polyphenolic flavonoid commonly found in fruits and vegetables, such as grapes, apples, blueberries, and onions [1, 2]. Pre-clinical studies that have explored the effects of quercetin have found it to demonstrate anti-inflammatory, antiviral, antioxidant, cardio-protective, anti-carcinogenic and neuroprotective properties, among many others [3–13]. The properties associated with quercetin have endorsed this dietary agent as a potential treatment for inflammatory diseases and cancer, as well as related conditions like cachexia [5, 12, 14, 15]. However, continued uncertainty surrounds the potential toxicity of quercetin which impedes its utilization in the clinic [4, 16–22].

Pre-clinical investigations have been conducted to evaluate the safety of quercetin as a dietary supplement. The data gathered in previous studies reported potential side effects of quercetin, including reduction in glutathione levels, increase in lactate dehydrogenase leakage, increase of the cytosolic free calcium concentration in rat lung epithelial cell line, and even death when co-administered with digoxin (cardiac glycoside) in pigs [20, 21]. Furthermore, quercetin exhibited carcinogenic activity in the form of treatment-related lesions in the kidneys, urinary bladder, and intestine in male rats after chronic consumption (58 and 104 weeks) of very high doses of quercetin (1,000 and 40,000 ppm) [17, 22]. While these studies reported adverse effects, there is a much larger literature base that has reported no adverse side effects [4, 5, 12, 14, 15, 18, 19, 23, 24]. Further, there also is a large body of evidence documenting efficacy of quercetin, including work done by our group, in the cancer, cancer therapy, and cancer cachexia domain. However, the contradictory evidence for and against the utilization of quercetin as a dietary compound continues to hinder the progression and development of quercetin as a promising treatment for cancer, and therapy-associated side effects [5, 15].

While many studies have evaluated quercetin for dietary consumption in rats [17, 18, 22, 23], only one study in mice has investigated the long-term effect of chronic quercetin use [19]. Given the potential for adverse effects associated with quercetin administration, it is vital to continue evaluating the potential toxicity of quercetin. To accomplish this, we conducted a sub-chronic study of quercetin toxicity. Male and female CD2F1 mice were fed a quercetin-containing AIN-76A diet over the course of 98 days. The diet was infused with three different doses of quercetin (equivalent to ~ 12.5 mg/kg, 25 mg/kg, 50 mg/kg of BW based on the food intake estimate) in order to determine potential toxicity at different concentrations. Behavioral tests, metabolic measurements, blood analysis, and histology were conducted to measure physiological and behavioral parameters related to the sub-chronic ingestion of quercetin.

## Methods

### Animals and diet

Male (*n* = 20) and female (*n* = 20) *CD2F1* (CDF1) hybrid mice (from Charles River Laboratory, Raleigh, NC)—a cross between Balb/cAnNCrl and DBA/2NCrl, making them ideal for safety and efficacy studies – were used in this study. Around 10 weeks of age mice arrived to our facilities and they were randomly assigned to one of four groups: Control (AIN76A-diet, *n* = 5 female, *n* = 5 male), Low Quercetin (~ 12.5 mg/kg of BW, *n* = 5 female, *n* = 5 male), Medium Quercetin (~ 25 mg/kg of BW, *n* = 5 female, *n* = 5 male), and High Quercetin (~ 50 mg/kg of BW, *n* = 5 female, *n* = 5 male). We selected these doses as they have been shown by our groups to have efficacy in models of cancer, cancer therapy, and cancer cachexia [5, 11, 12, 15]. Mice were housed 5 per cage with wood bedding and nesting material. To determine quercetin activities on behavioral and metabolic phenotyping, we used additional CD2F1 mice fed with AIN76A (*n* = 4 female and *n* = 4 male) and High Quercetin (50 mg/kg of BW, *n* = 4 female, *n* = 4 male). Mice were kept on a 12:12 h light/dark cycle, in a humidity and temperature-controlled room, and had ad libitum access to water and food. Animal handling and experiments were performed to minimize pain and discomfort. All procedures involving animals were reviewed and approved by the Institutional Animal Care and Usage Committee (IACUC) at the University of South Carolina (animal protocol number, 2410–101,524-081,920) performed in accordance with the American Association for Laboratory Animal Science.

Mice were accustomed to the AIN-76A diet (BioServ, Frenchtown, NJ, USA; catalog# F1515) for 4 weeks prior to any experimental procedure (from 10–14 weeks of age). At 14 weeks of age mice were introduced to quercetin-containing AIN-76A diet or maintained on AIN-76A diet for 98 days: Low Quercetin (BioServ, Frenchtown, NJ, USA; catalog# F7974, pink 1/2" pellets) contains 62 mg of Quercetin per kg of diet; Medium Quercetin (BioServ, Frenchtown, NJ, USA; catalog# F7975, blue 1/2" pellets) contains 125 mg of Quercetin per kg of diet; High Quercetin (BioServ, Frenchtown, NJ, USA; catalog# F7976, yellow 1/2" pellets) contains 250 mg of Quercetin per kg of diet. Quercetin was purchased from Sigma-Aldrich (St. Louis, MO, USA; catalog # Q4951). Body weight, food consumption, and water intake were measured weekly. Body composition, behavioral phenotyping, and metabolic assessment were examined monthly. Mice were euthanized (overdose of isoflurane) at 28 weeks of age (after 14 weeks of treatment); spleen, liver, heart, kidney, colon, fat, and muscles were dissected, weighed, and stored at -80 °C or fixed in 10% formaldehyde for further analysis.

### Body composition

Body composition was assessed at month 1, 2, and 3 after the initiation of the Quercetin-containing AIN-76 diets using dual-energy X-ray absorptiometry (DEXA) (Lunar PIXImus, Madison, WI, USA). Mice were briefly placed under gas anesthesia (isoflurane, 2%) and were assessed for bone mineral density (BMD), lean mass, fat mass, and body fat percentage.

### Behavioral and metabolic phenotyping

An additional cohort of sixteen CD2F1 mice (8-female and 8-male) were used for behavioral and metabolic phenotyping using the Prometion behavioral and metabolic cages (Sable System, Las Vegas, NV). A total of eight mice (4 female and 4 male) were fed AIN-76A diet and another eight mice (4 female and 4 male) consumed the High-Quercetin diet as stipulated previously in the animal and diet section. Behavioral and metabolic assessments were evaluated at month 1, 2, and 3 months after the initiation of the Quercetin-containing AIN-76 diet. Prior to data collection, mice were singly housed and acclimated for 3 days to the behavioral/metabolic cages and data were collected and analyzed for the subsequent 7 days. The behavioral/metabolic system consists of 16 cages each one of them equipped with water bottles, food hoppers, body mass habitat, multiplexed respiratory system, and BXYZ beam break activity monitor. All animals had ad libitum access to food and water. Food and water consumption, body weight, total activity, energy expenditure, respiratory quotient, animal ambulatory locomotion, and sleeping patterns during each 12:12-h light/dark cycle were collected and analyzed. The percentage of each animal’s total time engaged in eating, drinking, inside their habitat, and sleeping were calculated using Expedata and automated analysis scripts (Sable System International, Las Vegas, NV, USA).

### Blood panel analysis

A complete blood panel analysis was performed every month using the VetScan HMT (Abaxis, Union City, CA, USA) for determination of hematocrit (HCT), and hemoglobin (Hb), erythrocyte count, mean corpuscular hemoglobin, mean corpuscular volume, mean corpuscular hemoglobin concentration, total differential leukocyte counts (WBC), lymphocytes (LYM), monocytes (MON), neutrophils (NEU), and platelets (PLT).

### Clinical chemistry assessment

Clinical chemistry tests were used to assess liver function, cellular function, and carbohydrate metabolism. Specific determinations included: alanine transaminase (ALT) (Cayman Chemicals, Ann Arbor, MI, USA; catalog no. 700260), aspartate transaminase (AST) (Cayman Chemicals, Ann Arbor, MI, USA; catalog no. 701640), alkaline phosphatase (Cayman Chemicals, Ann Arbor, MI, USA; catalog#: 701,710), and creatinine (Cayman Chemicals, Ann Arbor, MI, USA; catalog no. 700460) according to manufacturer’s instructions. Prior to euthanasia, mice were fasted for five hours (light cycle). For blood glucose assessment in fasted mice, the tip of the tail was cut with scissors and blood glucose was assessed using a glucometer (Bayer Counter, New Jersey, US). All other analysis was carried out using plasma from blood collected from the inferior vena cava at euthanasia.

### Staining and histopathology

All tissues collected were stained with CAT hematoxylin (Biocare Medical, CA, USA; catalog no. CATHE) and Edgar Degas Eosin-Y (Biocare Medical, CA, USA; catalog no. HTE) also known as H&E staining. We used picro-sirius red stain kit (Abcam, Cambridge, MA, US; catalog no. ab150681) to evaluate tissue fibrosis. Histopathological evaluation of the colon, kidney, spleen, heart, gonadal fat, and liver sections of all animals of Control and treated with Low Quercetin, Medium Quercetin, and High Quercetin groups was performed blindly by a trained graduate student (P.C.) and a certified pathologist (I.C.). The specimens were evaluated for findings of inflammation, dysplasia, and fibrosis.

### Statistical analyses

Data were analyzed using Prism 8 statistical software (GraphPad Software, CA, USA). A One-Way ANOVA followed by Tukey’s multiple comparison test was used to determine differences between groups (Control, Low-Quercetin, Medium-Quercetin, and High-Quercetin). Two-Way Repeated Measure ANOVA with a Sidak’s multiple comparison test was used to assess differences in behavioral parameters. Analysis of covariance was used for metabolic parameters. Data are presented as the mean ± SEMs and the level of significance was set up at p ≤ 0.05.

## Results

### Fourteen weeks of quercetin feeding did not alter body composition

**Fig. 1:**
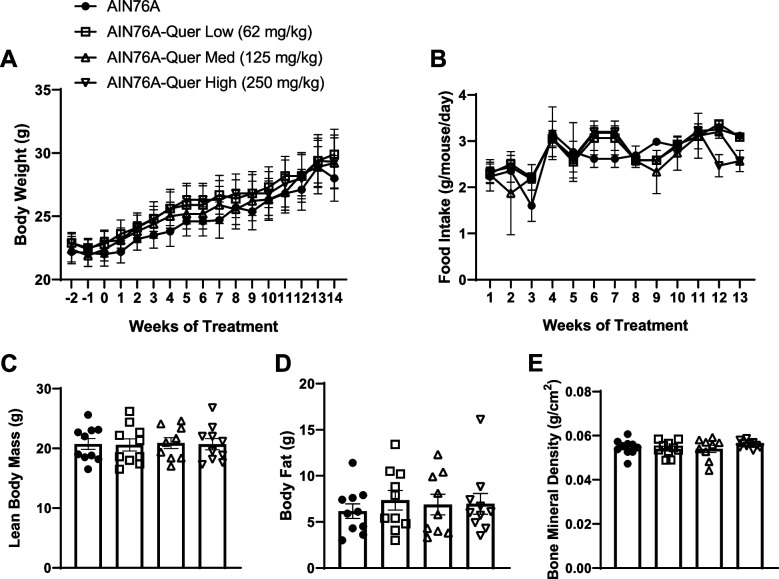
Three-month quercetin feeding did not impact body composition. Body composition was observed at months 1, 2 and 3 of quercetin feeding. Body weight (**A**) and food intake (**B**) of each treatment group was measured weekly. Lean body mass (**C**), body fat (**D**), and bone mineral density (**E**) were gathered via DEXA. Values were represented by mean ± SEM; *n* = 10 mice per group. One-way ANOVA followed by Tukey’s multiple comparison test *p < 0.05

The body weight of the mice was measured weekly after they were fed the AIN-76A purified diet and diets with various quercetin concentrations. Body composition of the mice was assessed via DEXA. While the body weight increased throughout the experiment, there were no significant differences in the body weight or food intake between the four groups (Fig. 1A-B). Lean body mass, body fat, and bone mineral density were gathered for each mouse at month 1 (data not shown), 2 (data not shown), and 3 (Fig. 1C-E). No significant differences were observed in lean body mass (Fig. 1C), body fat (Fig. 1D), and bone mineral density (Fig. 1E) at any time point for any of the quercetin doses versus the control group (AIN-76A). Further, there were no changes in organ weight (spleen, liver, heart, gonadal fat, mesenteric fat, kidney fat, gastrocnemius, quadriceps, and colon) and tibia length with sub-chronic administration of quercetin in the diet (Table. 1). To investigate metabolic risk associated with changes in body composition, we assessed fasting blood glucose at 14 weeks (i.e. 98 days) of quercetin feeding. Consumption of quercetin at various doses did not impact fasting blood glucose levels (Table. 1).

**Table 1:** Three-month quercetin feeding did not impact fasting glucose and organ weight

	**AIN76A**	**AIN76A-Quer Low (62 mg/kg)**	**AIN76A-Quer Med (125 mg/kg)**	**AIN76A-Quer High (250 mg/kg)**
Glucose (mg/dL)	98.1 ± 6.8	103 ± 3.8	99.1 ± 2.7	106 ± 4.9
Spleen (mg)	70.3 ± 2.2	74.7 ± 3.6	71.1 ± 3.4	75.8 ± 3.9
Liver (g)	1.14 ± 0.1	1.24 ± 0.1	1.23 ± 0.1	1.35 ± 0.1
Heart (mg)	140 ± 6.3	132 ± 5.8	141 ± 7.6	141 ± 8.6
Gonadal fat (mg)	811 ± 127	1057 ± 116	990 ± 146	1095 ± 118
Mesenteric fat (mg)	377 ± 75	463 ± 68	419 ± 67	528 ± 91
Kidney fat (mg)	351 ± 68	414 ± 80	365 ± 75	414 ± 92
Gastrocnemious (mg)	125 ± 6.3	124 ± 4.5	127 ± 4.5	128 ± 4.1
Quadriceps (mg)	180 ± 9.4	176 ± 6.3	179 ± 6.3	161 ± 8.1
Tibia Length (mm)	17.9 ± 0.1	18.1 ± 0.1	18.3 ± 0.1	18.1 ± 0.2
Colon (mg)	104 ± 2.2	108 ± 2.1	108 ± 2.0	106 ± 4.2

### Fourteen-weeks of quercetin feeding did not elicit changes in blood cell count

Complete blood count with differential was used to assess the sub-chronic effects of quercetin on blood cells. No significant differences were observed in WBC and RBC at any time point for any of the quercetin doses versus the control group AIN-76A (Table 2). No significant changes were observed in the different types of WBCs; LYM, MON, and NEU. The amount of HGB and HCT of quercetin fed mice were also similar to control mice fed the AIN-76A diet. Mean corpuscular volume (MCV), mean corpuscular hemoglobin (MCH), and mean corpuscular hemoglobin concentration (MCHC) were measured to determine the size of the RBC, amount of HGB per RBC, and amount of HGB per unit volume. Fourteen-week quercetin (i.e. 98 days) feeding does not present changes in MCV, MCH, and MCHC between treatment groups.

**Table 2:** Three-month quercetin feeding did not affect complete blood count

**Time-line**	**1-month**				**2-month**				**3-month**			
**CBC counts**	**AIN76A**	**AIN76A-Quer Low (62 mg/kg)**	**AIN76A-Quer Med (125 mg/kg)**	**AIN76A-Quer High (250 mg/kg)**	**AIN76A**	**AIN76A-Quer Low (62 mg/kg)**	**AIN76A-Quer Med (125 mg/kg)**	**AIN76A-Quer High (250 mg/kg)**	**AIN76A**	**AIN76A-Quer Low (62 mg/kg)**	**AIN76A-Quer Med (125 mg/kg)**	**AIN76A-Quer High (250 mg/kg)**
**RBC (10^12/l)**	10.2 ± 0.2	9.0 ± 1.0	8.7 ± 0.8	8.9 ± 0.4	9.3 ± 0.4	9.6 ± 0.2	9.7 ± 0.1	9.3 ± 0.2	10.5 ± 0.5	10.1 ± 0.5	9.99 ± 0.3	8.40 ± 0.9
**HGB (g/dl)**	17.5 ± 0.3	15.6 ± 1.7	14.7 ± 1.4	15.5 ± 2.4	16.2 ± 0.6	16.4 ± 0.3	16.3 ± 0.3	16.1 ± 0.5	18.2 ± 1.1	17.3 ± 0.9	17.1 ± 0.5	15.7 ± 0.7
**HCT (%)**	38.2 ± 1.7	35.6 ± 3.4	38.2 ± 0.7	36.8 ± 0.4	37.0 ± 1.4	38.4 ± 0.6	38.4 ± 0.4	37.4 ± 0.8	42.3 ± 2.4	40.8 ± 1.2	40.3 ± 1	37.1 ± 1.6
**MCV (fl)**	40.0 ± 0.1	40.1 ± 0.1	40.0 ± 0.1	40.4 ± 0.2	39.8 ± 0.1	40.1 ± 0.1	39.8 ± 0.2	40.2 ± 0.1	40.2 ± 0.2	40.3 ± 2	40.3 ± 0.2	40.2 ± 0.1
**MCH (pg)**	17.2 ± 0.2	17.2 ± 0.3	16.9 ± 0.3	17.3 ± 0.2	17.4 ± 0.1	17.1 ± 0.1	16.9 ± 0.1	17.2 ± 0.2	17.4 ± 0.3	17.0 ± 0.2	17.1 ± 0.1	17.0 ± 0.1
**MCHC (g/dl)**	43.1 ± 0.4	42.8 ± 0.7	42.2 ± 0.6	42.8 ± 0.5	43.7 ± 0.2	42.8 ± 0.3	42.5 ± 0.4	42.9 ± 0.6	43.1 ± 0.8	42.4 ± 0.5	42.5 ± 0.3	42.3 ± 0.2
**PLT (10^9/l)**	200 ± 21	252 ± 51	210 ± 38	331 ± 62	136 ± 23	250 ± 45	310 ± 58	330 ± 74	156 ± 24	116 ± 29	133 ± 44	91 ± 31
**WBC (10^9/l)**	11.1 ± 0.5	7.7 ± 1.1	8.8 ± 1.1	10.4 ± 2.1	11.1 ± 0.9	9.1 ± 1.2	7.9 ± 1.6	6.9 ± 0.8	9.3 ± 1.6	9.0 ± 1.0	9.9 ± 1.2	8.3 ± 1.3
**LYM (10^9/l)**	7.6 ± 0.4	5.0 ± 0.7	6.3 ± 0.7	7.2 ± 1.3	8.0 ± 0.7	6.9 ± 0.8	5.6 ± 1.1	5.1 ± 0.7	6.1 ± 1.0	5.4 ± 0.4	6.7 ± 0.9	5.0 ± 0.8
**MON (10^9/l)**	0.4 ± 0.2	0.4 ± 0.1	O.4 ± 0.1	0.2 ± 0.1	0.5 ± 0.2	0.5 ± 0.2	0.4 ± 0.2	0.2 ± 0.1	0.5 ± 0.1	0.3 ± 0.1	0.6 ± 0.1	0.2 ± 0.1
**NEU (10^9/l)**	3.1 ± 0.1	2.3 ± 0.4	2.1 ± 0.4	2.9 ± 0.6	2.6 ± 0.3	1.8 ± 0.3	1.9 ± 0.4	1.6 ± 0.2	2.7 ± 0.5	2.7 ± 0.5	3.0 ± 0.3	2.5 ± 0.3

### Analysis of behavioral and metabolic measurements

**Fig. 2:**
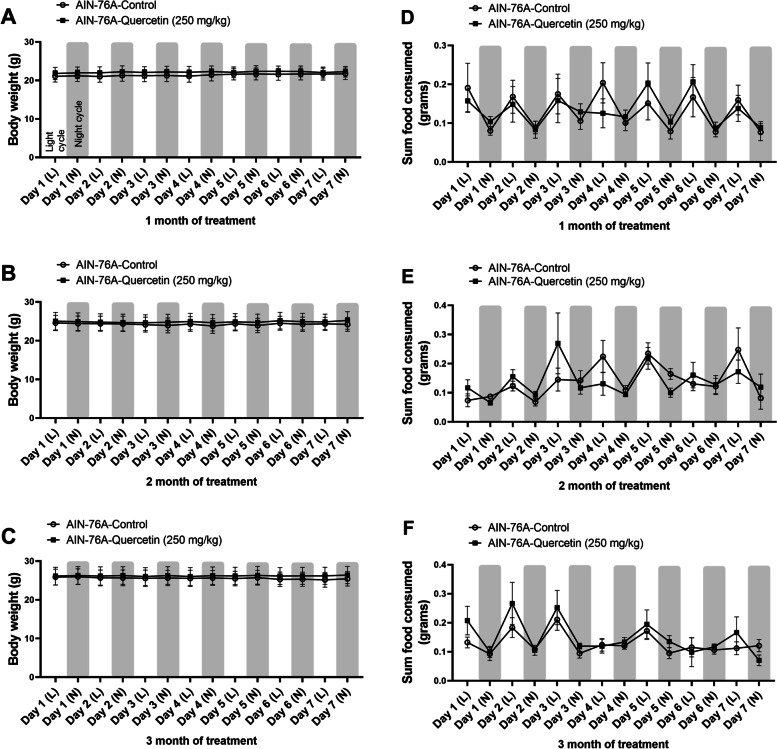
A 250 mg/kg quercetin-infused diet did not influence body weight or food consumption. Mice were singly housed in behavioral metabolic cages for 7 consecutive days intermittently (1, 2, and 3 months of feeding) over the course of the experiment and body weight (**A**-**C**) and food intake (**D**-**F**) were determined. Values were represented by mean ± SEM; *n* = 8 mice per group. Two-way RM ANOVA followed by Sidak’s multiple comparison test *p < 0.05

**Fig. 3:**
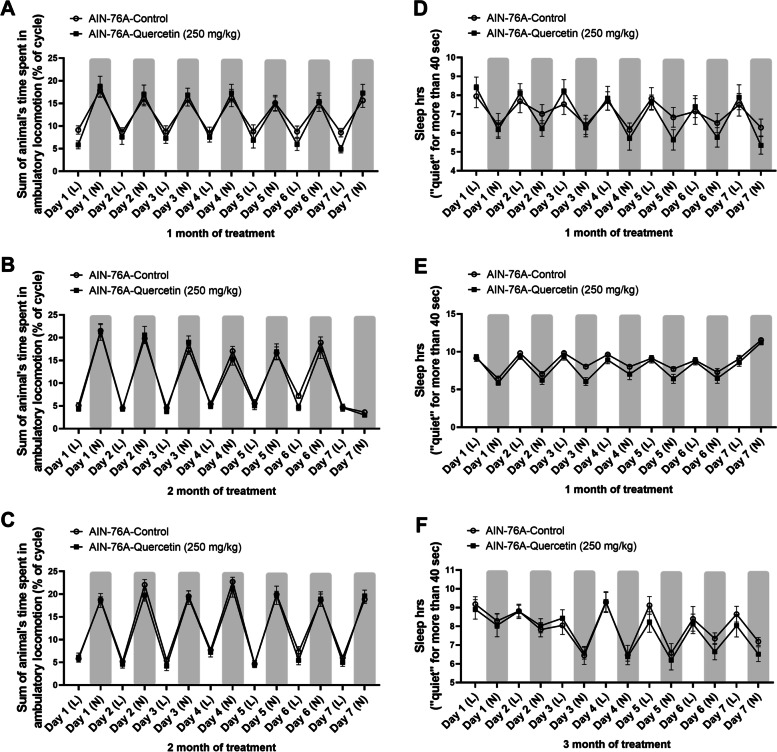
A 250 mg/kg quercetin-infused diet did not impact locomotion or sleep duration. Mice were singly housed in behavioral metabolic cages for 7 consecutive days intermittently (1, 2, and 3 months of feeding) over the course of the experiment and locomotion (**A**-**C**) and sleep duration (**D**-**F**) were assessed. Values were represented by mean ± SEM; *n* = 8 mice per group. Two-way RM ANOVA followed by Sidak’s multiple comparison test *p < 0.05

Additionally, we investigated the behavioral and metabolic profile of mice in the high dose of quercetin (~ 50 mg/Kg) given the relevance of these outcomes to assessing off-target effects of cancer and its therapies. Body weight, food consumption, locomotor activity, sleep, energy expenditure and respiratory quotient were assessed for seven consecutive days during the light and dark cycle (Table 3, Fig. 2, 3). There were no indications of body weight loss, decrease in food intake, nor change in ambulatory locomotion, sleep behavior, and indirect calorimetry outcomes between AIN-76A control mice and mice given the high dose of quercetin.

**Table 3:** A 250 mg/kg quercetin-infused diet did not alter metabolic phenotype

	**AIN76A**	**AIN76A-Quer High (250 mg/kg)**	***P* value**
Total EE (kcal/day)	9.87 (0.371)	9.75 (0.398)	0.8274
Energy intake (kcal/day)	8.11 (0.523)	7.50 (0.561)	0.4491
Resting EE (kcal/day)	0.34 (0.016)	0.32 (0.017)	0.4161
O2 Consumption (Light Cycle Average)	1.41 (0.053)	1.39 (0.057)	0.7967
CO2 Production (Light Cycle Average)	1.23 (0.039)	1.22 (0.042)	0.9255
Water Consumption (g/day)	1.93 (0.151)	1.71 (0.162)	0.3503
Avg RER (Light Cycle Average)	0.811 (0.009)	0.79 (0.019)	0.408
All Meters (day)	164.81 (6.78)	171.69 (14.80)	0.701

### The effects of quercetin on the liver

**Fig. 4:**
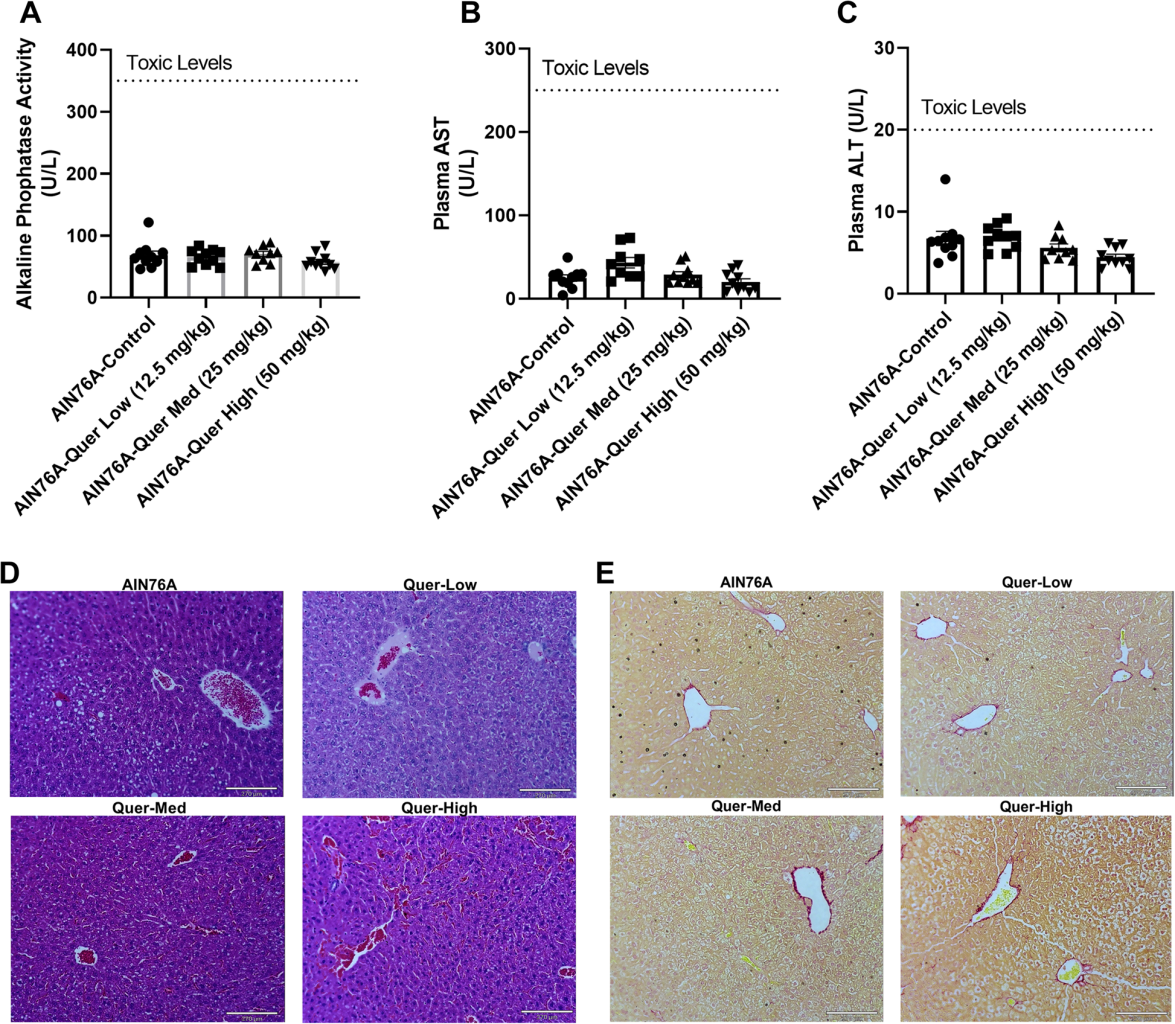
Consumption of quercetin did not elicit liver toxicity. Plasma alkaline phosphatase (**A**), AST (**B**), and ATL (**C**) were assessed after fourteen weeks of quercetin feeding. Livers were also assessed histologically (100X) using H&E (**D**) and picrosirius red stains (**E**). Values were represented by mean ± SEM; *n* = 10 mice per group. One-way ANOVA followed by Tukey’s multiple comparison test *p < 0.05

**Fig. 5:**
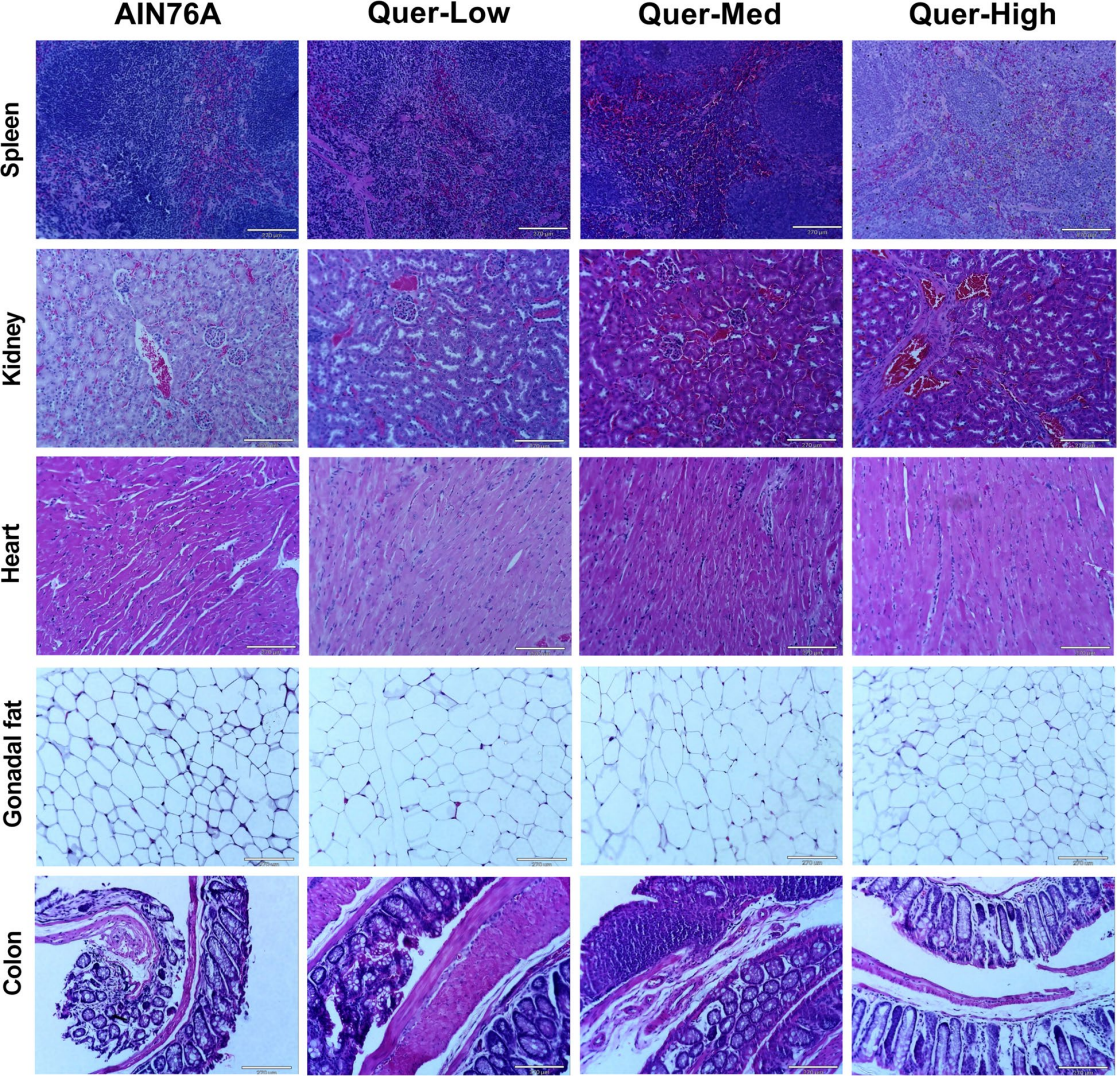
Quercetin-infused diets did not elicit pathological changes in multiple tissues. Representative H&E images of spleen, kidney, heart, gonadal fat, and colon

Clinical chemistry tests were utilized to determine the levels of plasma alkaline phosphatase, ALT, and AST. The blood used in this test was gathered from the inferior vena cava during euthanasia. Alkaline phosphatase activity did not show a significant difference between the four groups (Fig. 4A). Similarly, AST (Fig. 4B) and ALT (Fig. 4C) did not exhibit significant statistical differences between the control and the three quercetin treatment groups. The tests also showed that the levels of alkaline phosphatase, AST, and ALT were substantially below the levels considered toxic. H&E and picrosirius red staining (Fig. 5) were conducted on the collected tissues (spleen, liver, heart, kidney, colon, and gonadal fat). No significant histological changes were identified in the H&E-stained slides between control and treated groups (Fig, 4D and 5). In addition, no signs of fibrosis were found in tissues stained with picrosirius red in any of treated groups as compared to control (Fig. 4E).

## Discussion

Quercetin, a flavonoid commonly found in fruits and vegetables, has been endorsed as a possible treatment of inflammatory diseases and cancer, due to its anti-inflammatory, antiviral, antioxidant, cardio-protective, anti-carcinogenic and neuroprotective properties [3–5]. Despite this, the development of quercetin as a method of treatment is impeded by the uncertainties surrounding the toxicity of this natural compound. This study sought to investigate the possible toxicity of quercetin in a sub-chronic mouse model, using various doses of quercetin. We found that quercetin does not exhibit any negative side effects in mice at any of the doses used in the present study.

To determine the potential toxicity of quercetin, mice were given a quercetin-infused diet at various concentrations, over a span of 98 days. The data gathered throughout the experiment indicated that there were no discernible side effects associated with quercetin consumption. Previous studies that have observed the effects of quercetin have also found that there is no change in body composition associated with quercetin ingestion [25, 26]. Similarly, our data reflect this claim by exhibiting no significant difference in bone mineral density, lean mass, fat mass, and body fat percentage between the four groups. Previous studies have also investigated the effect of quercetin on organ weight, blood glucose, and blood count [27, 28]. Consistent with the data gathered in these studies, our data indicate that quercetin has no adverse effect on organ weight, blood glucose, and blood counts. Interestingly, in a study of quercetin treatment in patients with chronic obstructive pulmonary disease, found that quercetin may be linked to the normalization of fasting blood glucose levels [28]. This information further supports our data demonstrating that quercetin has no adverse effects on fasting glucose levels. However, it is important to continue researching quercetin’s effects on fasting glucose levels, as there has yet to be clear evidence regarding the method by which quercetin acts on blood glucose levels.

Analysis of quercetin’s effect on behavior and metabolic processes indicated that there are no adverse side effects associated with a quercetin-infused diet, at any dose. When observing body weight and food consumption, our results were similar to many previous studies, which found these factors were not altered when exposed to quercetin [29]. Evidence gathered in previous quercetin studies have possibly linked its use to increased endurance exercise capacity and performance [3]. Although we did not assess endurance capacity in our experiments, our findings indicate that quercetin does not interrupt spontaneous physical activity. While the mechanism by which quercetin improves performance has yet to be fully understood, the data presented in the current study indicates that quercetin does not negatively affect the movement of the mice in their home cage.

Data gathered from clinical chemistry tests were used to determine the levels of plasma alkaline phosphatase, ALT, and AST, common biomarkers for liver health. Analysis showed that there were no significant differences between the plasma enzyme levels between the control group and the 3 quercetin treatment groups. Similar results have been shown in previous studies that have observed quercetin’s toxicity, as well as its impact on the liver under various induced toxicities [17, 30].

We also sought to investigate the histology of the spleen, kidney, heart, gonadal fat, and colon due to previous studies reporting that quercetin exhibited carcinogenic activity in the kidneys, urinary bladder, and intestine in male rats after chronic consumption (58 and 104 weeks) of very high doses of quercetin (1,000 and 40,000 ppm) [17, 22]. In our study, we used a maximum of 250 mg/kg of quercetin in the diet (~ 250 ppm, 50 mg/kg of body weight, or 0.005% of quercetin) which does not show any histological changes or toxic effects. Indeed, other studies implementing prolonged exposure (104 weeks) of quercetin have shown no carcinogenic effects at doses ranging between 1.25–5% (1.25% = 15,000 mg/kg, 5% = 50,000 mg/kg) [24]. However, an increase in non-neoplastic polyps was observed in the intestines of male rats treated with 5% quercetin [24]. Therefore, we could extrapolate that the differences in quercetin carcinogenic activity might be due to the high concentration of quercetin (over 40,000 ppm or 40 mg/kg) and not to the chronic (104 weeks) exposure of quercetin. Whether this higher concentration of quercetin possesses biological significance remains unknown.

## Conclusions

This study focused on identifying the possible toxicity of quercetin and its effects on mice in a sub-chronic model. Our analysis did not indicate any adverse side effects associated with the administration of quercetin, at doses we have reported as efficacious in cancer, cancer therapy, and cancer cachexia models, over a 98-day period. This further suggests that quercetin is a safe dietary supplement at doses ranging between 62–250 mg/kg of the diet or 12.5–50 mg/kg body weight. The evidence obtained in this study supports quercetin’s potential as a treatment for inflammatory diseases and cancer, as well as related conditions such as cachexia. However, while this data is very promising, it is important to continue investigating into quercetin and in particular its long-term effects. Indeed, previous studies that have observed toxicity of quercetin were reported using chronic administration of this compound and at much larger doses [20]. The uncertainty surrounding quercetins toxicity will continue to delay its utilization as a treatment, making further research vital. Therefore, advanced pharmaceutical toxicology studies are necessary to facilitate the clinical administration of quercetin.

## Data Availability

All data generated during the current study are available from the corresponding author on reasonable request.

## Abbreviations

BW: Body weight
AP: Alkaline phosphatase
AST: Aspartate transaminase
ALT: Alanine transaminase
WBC: White blood cell
RBC: Red blood cell
HCT: Hematocrit
Hb: Hemoglobin
LYM: Lymphocytes
MON: Monocytes
NEU: Neutrophils
PLT: Platelets
